# Global Burden of Disease from Environmental Factors

**DOI:** 10.1146/annurev-publhealth-071823-105338

**Published:** 2024-12-17

**Authors:** Sierra N. Clark, Susan C. Anenberg, Michael Brauer

**Affiliations:** 1 School of Health & Medical Sciences, City St George’s, University of London, London, United Kingdom; 2 Milken Institute School of Public Health, George Washington University, Washington, DC, USA; 3 Institute for Health Metrics and Evaluation, University of Washington, Seattle, Washington, USA; 4 School of Population and Public Health, University of British Columbia, Vancouver, British Columbia, Canada

**Keywords:** burden of disease, environmental risk factors, global, review

## Abstract

Estimation of the disease burden attributable to environmental factors is a powerful tool for prioritizing environmental and pollution management and public health actions around the world. The World Health Organization (WHO) began estimating the environmental disease burden in 2000, which has formed the basis for the modern estimation approach conducted in the Global Burden of Disease, Injuries, and Risk Factor (GBD) study. In 2021, environmental and occupational risk factors in the GBD were responsible for 18.9% (12.8 million) of global deaths and 14.4% of all disability-adjusted life years (DALYs), led by ambient PM_2.5_ air pollution (4.2% DALYs, 4.7 million deaths) and household air pollution from the use of solid fuels for cooking (3.9% DALYs, 3.1 million deaths). Climate change exacerbates many environmental hazards, leading to increased disease burdens from heat, air pollution, vector-borne diseases, storms, and flooding. Other environmental risk factors not included in the GBD, such as poor indoor air quality, various chemical exposures, and environmental noise pollution, also significantly contribute to disease burden in many countries, though more efforts are needed to generate and integrate data resources for inclusion in global estimations.

## INTRODUCTION

Estimation of the disease burden attributable to environmental risk factors is a powerful tool for prioritizing environmental and pollution management and public health actions. Most individual research studies focus on a specific exposure and one or more health outcomes, which makes it challenging to evaluate the actual public health impact of a given exposure. Furthermore, as epidemiological analyses typically provide information on risks relative to a reference exposure, there is a gap in understanding the absolute risk—in other words, what the magnitude is of impact on health at a population scale. Disease burden estimation fills these gaps by estimating absolute health impacts of a given exposure, often in a larger context where exposures related to other (e.g., behavioral or metabolic) risk factors such as dietary intake or physical inactivity are also considered in a unified and comparative framework.

Disease burden has historically been quantified on the basis of mortality, which is relatively straightforward and quantified in many jurisdictions. Cause-specific mortality, in which deaths are categorized by causes according to international classification of disease (ICD) codes, provides an additional level of specificity but is less common given requirements for vital registration systems and medical death certification. However, even in instances where cause-specific mortality is accurately quantified, it provides an incomplete picture of health status throughout the life course.

In modern disease burden estimation, methods have therefore been developed to quantify the burden of disease using a standardized, time-based metric called disability-adjusted life years lost (DALYs). One DALY corresponds to one lost year of healthy life in a population. The DALY combines the burden from (*a*) mortality, in terms of years lost because of premature death due to disease [years of life lost (YLL)], and (*b*) morbidity, in terms of years of life adversely affected by disease [years of life lived with disability (YLD)], where morbidity impacts of disease are weighted by relative measures of severity given by the disability weight. The disability weight is measured on a scale of 0 to 1, with 0 indicating a state equivalent to full health and 1 being a state equivalent to death. For example, in the most recent iteration of the Global Burden of Disease, Injuries, and Risk Factor (GBD) study (34) (GBD 2021), mild diarrhea has a disability weight of 0.07, a migraine headache has a disability weight of 0.44, and acute schizophrenic state has a disability weight of 0.77. The sum of DALYs across a population over a period of time provides a measurement of the gap between actual health status and an ideal situation in which the entire population lives to an advanced age, free of disease and disability.

The population attributable fraction (PAF) is the proportional reduction in death or disease incidence that would occur if exposure to a risk, in this case an environmental hazard, was removed or reduced to an alternative level. PAF can be calculated by quantifying exposures and the relative risk (RR) at a given level of exposure and comparing this quantity to the RR at a counterfactual or theoretical minimum risk exposure level (TMREL). The TMREL may be zero for exposures such as secondhand smoke, where it is theoretically possible to eliminate exposure, or may be nonzero, for example in the case of ambient ozone air pollution where a natural background concentration is always present (Table 1). The PAF, the proportion of a specific disease that can be attributed to exposure, is then multiplied by the underlying burden of the disease (quantified by deaths, DALYs, YLDs, and/or YLLs) in a given population and time period to quantify the attributable disease burden.

## EVOLUTION OF ENVIRONMENTAL DISEASE BURDEN ESTIMATION

The World Health Organization (WHO) has periodically estimated disease burden related to environmental factors, with the first major analysis, the comparative risk assessment (CRA) (29), also referred to as GBD 2000, published as part of the World Health Report 2002 (84). This analysis included estimates of disease burden attributable to unsafe water, sanitation, and hygiene (handwashing) (WaSH), urban air pollution, indoor smoke from solid fuels, lead exposure, and climate change. For each of these risk factors, attributable burdens of one or more specific diseases were estimated quantitatively. This analysis formed the basis for the modern disease burden estimation approach that is conducted in the GBD study. In GBD 2000, estimates of exposure to environmental risk factors were based mainly on surveys and RRs estimated from meta-analyses of the available literature. In 2006, the WHO developed an analysis (64) using markedly different methodology based on standardized surveys of expert opinion to estimate attributable fractions to a broader range of environmental risk factors, including those from the 2002 World Health Report as well as occupational exposure to noise (hearing loss), housing risks, chemicals, recreational environments, water resource management, land use and the built environment, radiation, and climate change. Notably, this analysis did not estimate exposure, nor did it attribute the burden to the specific environmental risks. In total, this analysis estimated that 24% of the global disease burden and 23% of all deaths were attributable to this broad group of environmental factors (including occupational exposures). An updated analysis conducted by the WHO for the year 2016 (66) combined the expert opinion approach to estimate attributable fractions with CRA methods (based mainly on those used in GBD 2010) and arrived at a similar conclusion that 23% of global deaths were attributable to the included environmental (and occupational) risk factors. Sixty-eight percent of these attributable deaths were estimated with the evidence-based CRA methods, while the remainder were based on expert opinion of attributable fractions.

The GBD study is the most comprehensive analysis of the environmental burden of disease and the only one that is regularly updated every ~1–2 years. Analysis is undertaken by the Institute for Health Metrics and Evaluation (University of Washington, Seattle), together with an international collaborative network including, in 2024, more than 13,000 individuals from 163 countries and territories. Arising from the CRA above, the first major release was GBD 2010, followed by releases every 1–2 years. In each cycle, the full time series from 1990 is re-estimated with improved and expanded methodology, new input data, and expansion of included risk factors and diseases. In its most recent iteration, GBD 2021, the analysis includes estimation of the disease burden attributable to 13 environmental risk factors (Table 1) for the years 1990–2021, differentiated by age and sex for 204 countries and 811 subnational locations globally. While the CRA methodologies have been mainly applied globally, there is increasing interest from regional, national, and local governments in conducting their own disease burden estimates using local exposure data or RR information derived from local studies, estimating disease burden relative to a proposed environmental standard (and not a TMREL), and/or applying the methodology to environmental risk factors not currently included in the GBD.

In the CRA and GBD methodology, estimation of the burden attributable to risk factors requires four components. First, the distribution of the exposure across a population must be estimated. Methods to estimate exposure range from the use of biomarkers (e.g., blood lead) to geospatial satellite–derived estimates (e.g., ambient PM_2.5_ and nitrogen dioxide air pollution) to geospatial regression models incorporating survey data or environmental measurements [e.g., drinking water, sanitation, household air pollution (HAP), ozone, radon]. These population exposure estimates are then integrated with RR relationships derived from meta-regressions of systematic literature reviews of randomized trial and epidemiological studies and the TMREL to calculate disease-specific PAFs (Equations 1 and 2). PAFs are then multiplied by the underlying level of the disease within a population (differentiated by location, age, sex) of interest to derive the disease burden attributable to each risk factor. This estimate addresses the question, What would the health gain be if all exposure was reduced to the counterfactual level? In each iteration of the GBD, the entire time series of estimates (annually from 1990) is recalculated with the most recent methodology and input data to be internally consistent. Although secondhand smoke is described as a behavioral (and not environmental) risk factor in the GBD hierarchy, we include it here, given that it may be considered an important component of indoor air pollution.

Binary exposure:

1. $$PAF=\frac{p\times (RR-1)}{p\times (RR-1)+1}$$

Continuous exposure:

2. $$PAF=\frac{\sum_{i=1}^{n}p_{i}\times (RR_{i}-1)}{\sum_{i=1}^{n}p_{i}\times (RR_{i}-1)+1}$$

In 2021, in total the environmental and occupational risk factors (excluding secondhand smoke) were responsible for 18.9% [12.8 million (M)] of all deaths and 14.4% of all DALYs globally (9). Ambient fine particulate matter (PM_2.5_) air pollution was the leading global environmental risk factor accounting for 4.2% of all DALYs and 4.7 M deaths followed by HAP (3.9%, 3.1 M). Between 2000 and 2021 (2010–2021), DALY rates standardized to a population of 100,000 people (DALYs per 100,000) decreased globally for most of the environmental risk factors. For example, there were 43% (−31% for 2010–2021), 60% (−45%), and 64% (−49%) decreases in attributable DALY rates for HAP, unsafe water, and unsafe sanitation, respectively. In contrast, DALY rates increased by 41% (22%) for ambient particulate matter air pollution, 45% (25%) for ozone, 32% (13%) for bone lead (in relation to cardiovascular disease), and 9.6% (10%) and 8.5% (−34%) for low and high temperatures, respectively.

A key aspect of interpreting the global disease burden from environmental risk factors is the interaction between exposure and population demographics. As the disease burden attributable to environmental risk factors is the product of the PAF (a function of exposure and RR) and the underlying rate of specific diseases causally related to that risk factor, as these rates increase—for example, through aging—attributable burden will also increase. Among the environmental risk factors—unsafe water, unsafe sanitation, handwashing, HAP from the use of solid fuels for cooking, nitrogen dioxide, and secondhand smoke—all had decreasing global exposures and decreasing attributable burden between 2000 and 2021. For these risk factors, programs to reduce exposure should be maintained, especially given that substantial attributable health burdens remain, for example, in the cases of unsafe water and HAP. Lead exposure falls into a separate category in which decreases in exposure have been insufficient to counteract the impact of population aging, leading to an increased estimated attributable burden. In the case of lead, this increase in attributable burden is due to the relatively underappreciated impact of cumulative lead exposure on cardiovascular disease (63), responsible for 1.5 M attributable deaths globally in 2021, a figure that is likely to grow in subsequent years as populations continue to age and develop cardiovascular disease. Another group of environmental risk factors includes high temperatures (covered in the section below titled Climate Change), ambient PM_2.5_, and ozone, where exposure has increased globally and, in combination with increasing underlying disease rates related to aging, leads to increasing attributable burden. For these risk factors, exposure reduction should be prioritized.

## CLIMATE CHANGE

There is intense interest among the global health community in estimating the health impacts of a changing climate, a global environmental threat with a worrisome future trajectory. Climate change can magnify hazards posed by other environmental risk factors, such as heat, air pollution, vector-borne diseases, storms and flooding, and wildfires (16). These amplifications are already occurring at present and are expected to worsen in the future as the effects of climate change grow and compound. The general approach for modeling disease burdens associated with climate change is to (*a*) simulate the effects of greenhouse gases (GHGs) and associated climate change on meteorological and other conditions (e.g., sea level rise) in physical climate models; (*b*) estimate impacts of meteorological and other conditions on the magnitude and spatial distribution of environmental risk factors; and (*c*) calculate impacts of environmental risk factors on human mortality and morbidity. Disease burdens associated with climate change are often calculated by comparing scenarios reflecting different degrees of GHG emissions and climate change, such as the representative concentration pathways (RCP), and sociodemographic scenarios, such as the shared socioeconomic pathways (SSP) (57). Ensembles of multiple climate models and 10–20-year averaging periods are often used to account for uncertainty and interannual variability.

Impacts of climatic conditions on environmental risk factors can be estimated using statistical and process-based approaches. Statistical approaches develop associations between climatic variables and environmental risk factors using historical data. Historical associations are then combined with scenario-specific climate model outputs to project future changes in environmental risk factors (e.g., 2). Process-based modeling provides a more mechanistic understanding of these relationships. The outputs from the climate models (e.g., temperature, precipitation) are input into geophysical, ecosystem, or other models to estimate exposure to environmental risk factors (e.g., 61). Exposure to the risk factor can be translated into cases of human mortality and morbidity using epidemiological exposure–response relationships, similar to the common approach used for other environmental risk factors and described elsewhere in this review.

In 2014, the WHO estimated that climate change was projected to increase global deaths by 250,000 annually between 2030 and 2050 (86). These include 38,000 additional deaths due to heat exposure among elderly people, 48,000 due to diarrhea, 60,000 due to malaria, and 95,000 due to inadequate childhood nutrition in 2030. The report indicated that these were underestimates because several important causal pathways were not quantified. The WHO report remains the only attempt at using a CRA framework, considering multiple climate-sensitive environmental risk factors and multiple health outcomes, on a global scale. While focusing only on the United States, the US Environmental Protection Agency (EPA) also considers multiple health damages from climate change, including through heat, wildfires, dust, ozone, and aeroallergens, in its estimates of the societal damages from climate change (38). Its report finds that health damages from climate change are larger than any other sector included, driven largely by climate-related changes in temperature, ozone, and ambient PM_2.5_.

In 2019, the GBD study introduced nonoptimal temperature as a risk factor related to climate. This risk factor is subdivided into low and high temperatures based on location-specific deviations from location-specific all-cause minimum mortality temperatures. High temperature thus captures the direct impact of increased temperatures resulting from meteorologic variation and influenced by climate change. Consistent with the literature, the disease burden attributable to low temperatures substantially exceeds that from high temperatures; however, increases in high temperature exposure from 1990 to 2021 (0.5% annual rate of change in the summary exposure value) have exceeded any decrease in low temperature exposure (−0.1%). Indirect impacts of climate change have also been associated with health impacts but are only in the initial stages of being added to the GBD. Among the more prominent indirect impacts are those due to floods and storms, malaria and dengue due to changes in areas supporting vector survival, and impacts on measures of undernutrition due to climate impacts on local agricultural production and food security. Beginning in GBD 2021, the GBD has also routinely included forecasted disease burden (33). In this context, a reference temperature scenario is compared against a more optimistic scenario of emissions reductions based on RCPs and SSPs. The difference between these two trajectories can then be estimated as the future disease burden attributable to warmer temperatures resulting from climate change. These future trajectories also incorporate reductions in air pollution as a result of GHG mitigation efforts under the more optimistic scenario.

Unmitigated climate change is expected to exacerbate ground-level ozone and fine particulate matter (PM_2.5_), the two largest contributors to the global burden of diseases from ambient air pollution. Climate change enhances conditions for ozone to form and increases wildfire smoke and may also increase soil dust, contributing to PM_2.5_. Studies in the United States and Europe show that climate change also lengthens the pollen season. While no global scale assessment of the burden of disease from climate change has incorporated effects on air pollution, the US EPA incorporates ozone, wildfire smoke, dust, and pollen into its efforts to characterize the many societal damages from climate change (38). Results show that, among all societal damages from climate change, the health burden associated with climate-sensitive ozone and PM_2.5_ are among the top five in the United States in terms of the overall value of the damages. Studies expanding these estimates to the global scale also estimate large air pollution–related mortality burdens from climate change, with PM_2.5_ impacts outweighing ozone impacts (30, 74). These air pollution-related mortality burdens under future climate change may be further amplified by population aging (14, 91). From the perspective of mitigation, however, actions taken to reduce GHG emissions under the SSP scenarios are expected to lead to large reductions in levels of ozone and PM_2.5_ and their attributable disease burden (33, 81).

Climate change is expected to expand the suitable habitat for disease-carrying vectors such as mosquitoes and ticks, which transmit infectious diseases, including West Nile virus, dengue, Zika virus, chikungunya, and malaria (70). Considering just climate change, one analysis suggests that the climatically suitable period for malaria might increase by 1.6 months in areas of Africa, the Eastern Mediterranean, and the Americas, and the climatically suitable period for dengue might increase by 4 months in areas of the Western Pacific and Eastern Mediterranean regions (21). As a result, 4.7 billion additional people could be at risk of both diseases by 2070 relative to 1970–1999. However, the number of people contracting these vector-borne diseases may not increase proportionally. Many factors beyond climate change, including other global change processes, affect disease transmission and changes in case rates in different locations, and some may counteract each other (31). Disease spread and severity are also influenced by human and animal mobility, control measures, health services, and access to effective medications, among other factors (11). Incorporating vector-borne disease into global burden of disease assessments will need to consider these complexities, as well as potential adaptation measures that avoid exposure and protect health.

Other aspects of how climate change is expected to influence health are more nascent and are not covered in detail here. Flooding, storms, and sea level rise contribute directly and indirectly to loss of life and livelihood. Extreme weather events are associated with acute effects such as injuries, poisonings, wounds, gastrointestinal disease, infections, and mold, as well as longer-term mental health effects (23). Food security and undernutrition are also major concerns; consider the vast scale of the current problem, with an estimated 735 million people facing hunger in 2022 and potential exacerbation by climate change (70). Climate change may reduce crop yields, reduce the labor capacity of agricultural workers, disrupt the food security of people dependent on marine resources, and result in instability in food supply chains. Depression, anxiety, and other mental health outcomes, driven by both ecoanxiety and mental health impacts of climate-sensitive environmental risk factors, are also important to consider (20). Several of these risk factors can occur together, resulting in compound events and synergistic effects; for example, wildfires can increase flood risks, debris flow, and drinking water contamination (75). Methods and data may not yet be available to support inclusion of these important risk factors in global burden of disease assessments.

Comprehensively capturing the public health consequences of future climate change is critical to developing global mitigation and adaptation responses that protect public health and reduce the associated disease burden, as others have argued (13). Doing so requires further developing the evidence base for the multiple pathways through which climate change will impact public health, collaborating across disciplines to pull those pathways into global burden of disease estimates, and building approaches to account for possible adaptation scenarios.

## OTHER ENVIRONMENTAL RISK FACTORS

While the environmental risk factors included in the GBD have expanded since the original CRA—for example, the addition of ambient ozone, nitrogen dioxide, and high and low temperatures—other environmental risk factors may make meaningful and even large contributions to disease burden globally and/or in specific locations or to specific populations. For example, studies have highlighted the importance of environmental noise, UV radiation, pesticides, and other chemical exposures (32, 73). The GBD has recently introduced a quantitative evidence evaluation approach, the burden-of-proof risk function methods, which can be used to assess the strength of evidence for RR associations as an initial step toward quantifying disease burden (44, 92). One challenge with including additional risk factors in the GBD, given its regular updates, is that continuous updates of exposure data and new information informing RRs leading to expanding data requirements are needed. Meeting this need is particularly challenging for environmental risk factors where exposures may vary dramatically from one year to the next (e.g., temperature during an El Niño year). Still, regional or national analyses or those conducted by specific research groups can suggest the potential importance of additional risk factors not included in the GBD study and provide methodologic approaches that may be expanded globally. We highlight several examples below.

### Environmental Noise

The epidemiological and mechanistic evidence of the nonauditory health effects of long-term exposure to environmental noise has grown over the past decade, particularly with regard to road, rail, and aircraft transportation sources (10, 18, 27, 35, 40, 58, 76, 78, 83). Nighttime exposures are associated with disturbed sleep (76), and daytime exposures are associated with the disturbance of activities and communication, leading to adverse outcomes such as being highly annoyed and impaired cognition in both children and adults (17, 18, 25, 35, 58). Exposures throughout the day and night are also associated with cardiometabolic disease incidence [e.g., ischemic heart disease, heart failure, stroke, and type 2 diabetes (25, 51, 67, 71, 77, 83)], as a result of sustained physiological stress responses (e.g., from chronic activation of the sympathetic nervous system and the hypothalamic-pituitary-adrenal axis), which is further mediated by impaired sleep (27, 58, 59, 77). Recent studies also provide robust evidence of associations with all-cause mortality (25). Most of the epidemiological evidence comes from studies of road-traffic noise from European countries, where disease assessments of noise burden have, for the most part, been carried out (1, 24, 26, 36, 41, 45, 90).

In 2005, the WHO Regional Office for Europe began a process to quantify the burden of disease from environmental noise; in its 2011 report, the organization found that in Western European countries, at least 1 million DALYs were lost annually from traffic-related noise exposures, with sleep disturbance and annoyance accounting for most of the burden (90). In addition, since 2002, all European Union member states are required, through the Environmental Noise Directive (END), to prepare and publish maps for noise levels from major roads, railways, and airports and urban agglomerations above certain thresholds [A-weighted decibels (dBA)] every five years (28), allowing the European Environment Agency (EEA) to coordinate regular regionwide and country-specific burden of disease assessments (24). The EEA estimated that, due to environmental noise exposures in the European Territory in 2017, high annoyance (90 DALYs per 100,000), high sleep disturbance (80 DALYs per 100,000), and ischemic heart disease incidence (30 DALYs per 100,000) were major contributors to the environmental disease burden, particularly in urban areas. Furthermore, cognitive impairment affected ~12,400 children in total from aircraft noise exposures (24). Compared with GBD study estimates in the same region and time period (43), the total number of DALYs lost in Europe from transportation noise exposures (~1 million) were about 12 times lower than DALYs from ambient PM_2.5_ air pollution but 3.7 times higher than DALYs from high temperatures and 2.7 times higher than DALYs from ambient ozone air pollution. Since the 2020 EEA report, the health risk assessment methodology has been updated (in 2023) (25), and new estimates will be forthcoming.

Subnational assessments have also been conducted in several European countries (1, 6, 41, 45). For instance, in Nordic countries, DALYs in capital cities ranged from 329 to 485 per 100,000 people for road traffic noise and from 44 to 146 for railway noise exposures (1). A recent study in England also demonstrated significant spatial variations across small geographic areas in the health burdens attributable to road, rail, and aircraft noise exposures (45). These studies and others have also shown that disease burdens estimated with END noise mapping data may still be underestimated, as noise exposures below certain levels (e.g., <50 dBA L_night_, <55 dBA L_den_) or from minor sources (e.g., local roads) may not be reported (1, 41, 45).

The estimation of environmental noise–related disease burdens is currently limited to areas with spatially refined noise exposure data (most of which are in Europe), posing a challenge for scaling up a global assessment. Several studies have estimated environmental or road-traffic noise with measurements or models in some low- and middle-income country (LMIC) cities, showing in many cases that daytime and nighttime levels (in dB) exceed what has been modeled in European cities (15), and thus health burdens attributable to noise may be substantial in these regions, though currently unknown. Efforts are ongoing to identify and utilize data sources that could model global exposures to road-traffic noise for burden of disease applications (62). As the epidemiological evidence base develops, future burden of disease assessments may also indicate an expanded list of health outcomes associated with transportation noise, potentially including childhood behavioral problems, adult mental health (e.g., depression), dementia, and tinnitus (25, 72, 77).

### Indoor Air Pollution

People spend most of their time, on average, indoors (~80–90% in industrialized nations) (50). Burden of disease assessments of specific air pollutants common indoors (37) are distinct from those of HAP, which is concerned with PM_2.5_ pollution from solid fuel burning for cooking, heating, and/or lighting. To date, relatively few burden of disease assessments of indoor air pollution (IAP) or indoor air quality have been carried out (37, 85) due to the challenges of capturing long-term and representative exposure estimates indoors and the limited number of associated epidemiological studies tracking disease development or mortality over time (37, 50, 60, 68). For some pollutants common both indoors and outdoors (e.g., PM_2.5_ and NO_2_), debate is ongoing as to whether RRs derived from epidemiological studies where air pollution was monitored or modeled outdoors can be transferred to an indoor exposure microenvironment (22).

One of the first projects to apply environmental burden of methodology to IAP was the pan-European Environmental Burden of Disease in European countries project published in 2014 (36) and the follow-up HealthVent study published in 2016 (3). The HealthVent study estimated that across 26 European countries, IAP exposures were associated with 2.1 million DALYs per year (~400 per 100,000 people). The burden was dominated by cardiovascular diseases and from PM_2.5_ (outdoor origin, 62%; indoor origin, 16%), while carbon monoxide (CO), secondhand smoke, dampness, bioaerosols, radon, and volatile organic compounds together contributed 22% to the total burden (3). Higher levels of DALYs were found in Eastern European countries due to the high contribution of outdoor sources of air pollution (highest in Bulgaria, followed by Hungary, Romania, and the Czech Republic). The lowest DALYs from indoor exposure to air pollution were estimated in Sweden.

Several national IAP burden of disease assessments have also been undertaken around the world, such as in European countries (8, 19), the United States (55), Australia (47), New Zealand (69), and China. The Chinese Burden of Disease Attributable to Indoor Air Pollutants project (52, 53), initiated in 2017, made quantifications for numerous indoor air pollutants across China’s 31 provinces and found that indoor exposures to PM_2.5_ contributed the largest annual health burden [3,270 DALYs per 100,000 (~half of PM_2.5_ of indoor origin)], followed by CO (182 DALYs per 100,000), radon (91 DALYs per 100,000), benzene (68 DALYs per 100,000), nitrogen dioxide (65 DALYs per 100,000), ozone (62 DALYs per 100,000), SO_2_ (60 DALYs per 100,000), formaldehyde (54 DALYs per 100,000), toluene (21 DALYs per 100,000), and p-dichlorobenzene (9 DALYs per 100,000) in 2017 (54). This study also reported that the overall DALYs attributable to IAP in China were five times greater than those reported for European countries and twice those for the United States. This relative difference was driven largely by the DALYs for indoor PM_2.5_ in China (54).

### Chemical Exposures

Exposure to chemicals occurs every day through multiple routes such as inhalation, ingestion, dermal absorption, and in utero as many chemicals can cross the placenta. Chemical manufacturing is also on the rise, with approximately two-thirds of chemical production in LMICs (32, 48, 82). Given that only a small fraction of manufactured chemicals have been adequately tested for safety and toxicity, current quantified disease burdens are likely underestimated, particularly in LMICs, where data on chemical exposures are also limited (32). Based on the evidence available, the WHO created a list of the top ten chemicals of public health concern, which include lead, mercury, hazardous pesticides, air pollution, arsenic, asbestos, benzene, cadmium, dioxin, and inadequate/excess fluoride (88). Furthermore, the GBD study, the WHO, and the *Lancet* Commission on Pollution and Health have produced a series of estimates over the years quantifying and reporting on burdens of disease from chemical exposures (48, 65, 87, 89). The most recent report from the WHO (89), which includes some estimates from the GBD study, reported that 32 (male) and 15 (female) deaths per 100,000 people from noncommunicable diseases were attributable to chemical exposures in 2019 and that 3 (male) and 2 (female) deaths per 100,000 people were attributable to chemical-related injuries. Excluding the effects of general ambient air pollution, which is considered separately in the GBD study, chemical exposures were estimated to cause 2 million deaths (3.6% of total deaths) and 53 million DALYs (2.1% of total DALYs) from poisonings, self-inflicted injuries, congenital anomalies, cardiovascular diseases, chronic kidney diseases, idiopathic intellectual disability, cancers, pneumoconiosis, and chronic obstructive pulmonary disease (note that there is some overlap with GBD study estimates). The health and economic burdens of endocrine disruptor chemicals (EDC) have also been quantified in several regions and countries around the world (4, 5, 12, 56, 79). In Europe, an expert panel (7, 39, 42, 49, 79, 80) using epidemiological and toxicological evidence determined that there was probable EDC causation for IQ loss and associated intellectual disability, autism, attention-deficit hyperactivity disorder, endometriosis, fibroids, childhood obesity, adult obesity, adult diabetes, cryptorchidism, male infertility, and mortality associated with reduced testosterone, resulting in a median health-related cost of €163 billion annually in Europe (80). Since this work was published in 2016, the evidence base has grown and strengthened for several outcomes, including low birth weight (46).

## CONCLUSIONS AND COMPARISONS

**Figure 1:**
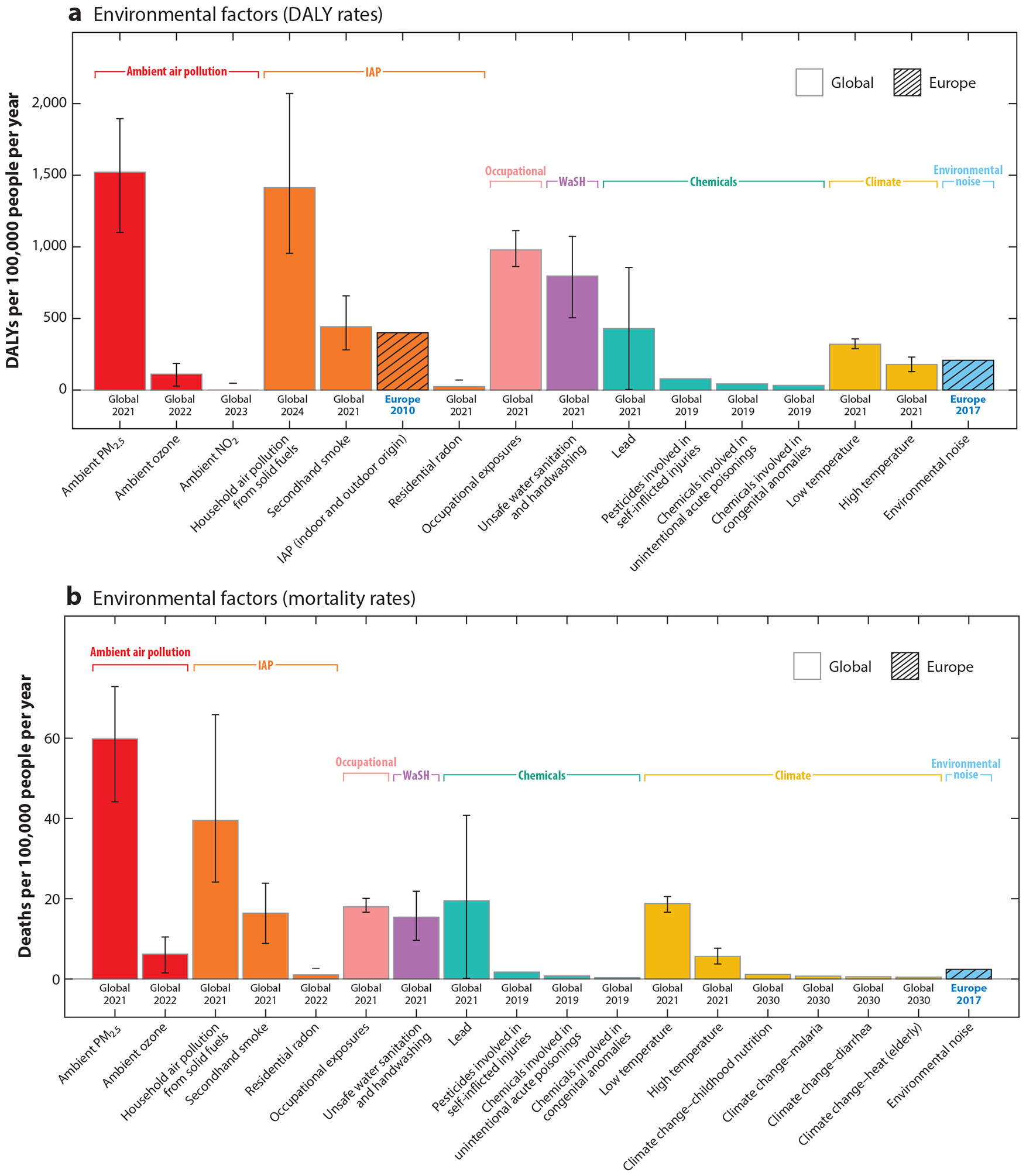
Annual (*a*) DALYs lost and (*b*) deaths per 100,000 people (all ages) attributable to environmental risk factors covered within this review. DALY and mortality rates from ambient air pollution (*red*) and IAP (*orange*) are sourced from the GBD 2021 project data (9), although DALY rates attributable to IAP (from indoor and outdoor origin) were estimated for 26 European countries for 2010 from the 2016 HealthVent study (3) (mortality not reported). DALY and mortality rates for the occupational exposures (*pink*) and WaSH (*purple*) categories are from GBD 2021 (9). The chemicals (*turquoise*) category includes DALY and mortality rates attributable to lead exposures from GBD 2021 and additional chemical exposures from a WHO 2021 report (89). The climate (*yellow*) category includes DALY and mortality rates attributable to low and high temperatures from GBD 2021 (9) and projected additional mortalities due to climate-sensitive diseases/risk factors for the year 2030 from the WHO 2014 (86) (mortality impacts only). The environmental noise (*blue*) category includes environmental noise–DALY and mortality rates attributable to exposures from road, rail, aircraft, and industrial sources, estimated for 33 countries in the EEA (not including Turkey) for 2017 as reported in the EEA 2020 report (24) (mortality was estimated with respect to only ischemic heart disease). When standardized population rates were not given, total numbers were standardized by the estimated global or regional population for the corresponding years. Uncertainty intervals are included where available. Abbreviations: DALYs, disability-adjusted life years; EEA, European Economic Area; GBD, Global Burden of Disease; IAP, indoor air pollution; PM_2.5_, particulate matter with diameters ~2.5 μm and smaller; WaSH, unsafe water, sanitation, and hygiene; WHO, World Health Organization.

**Figure 2:**
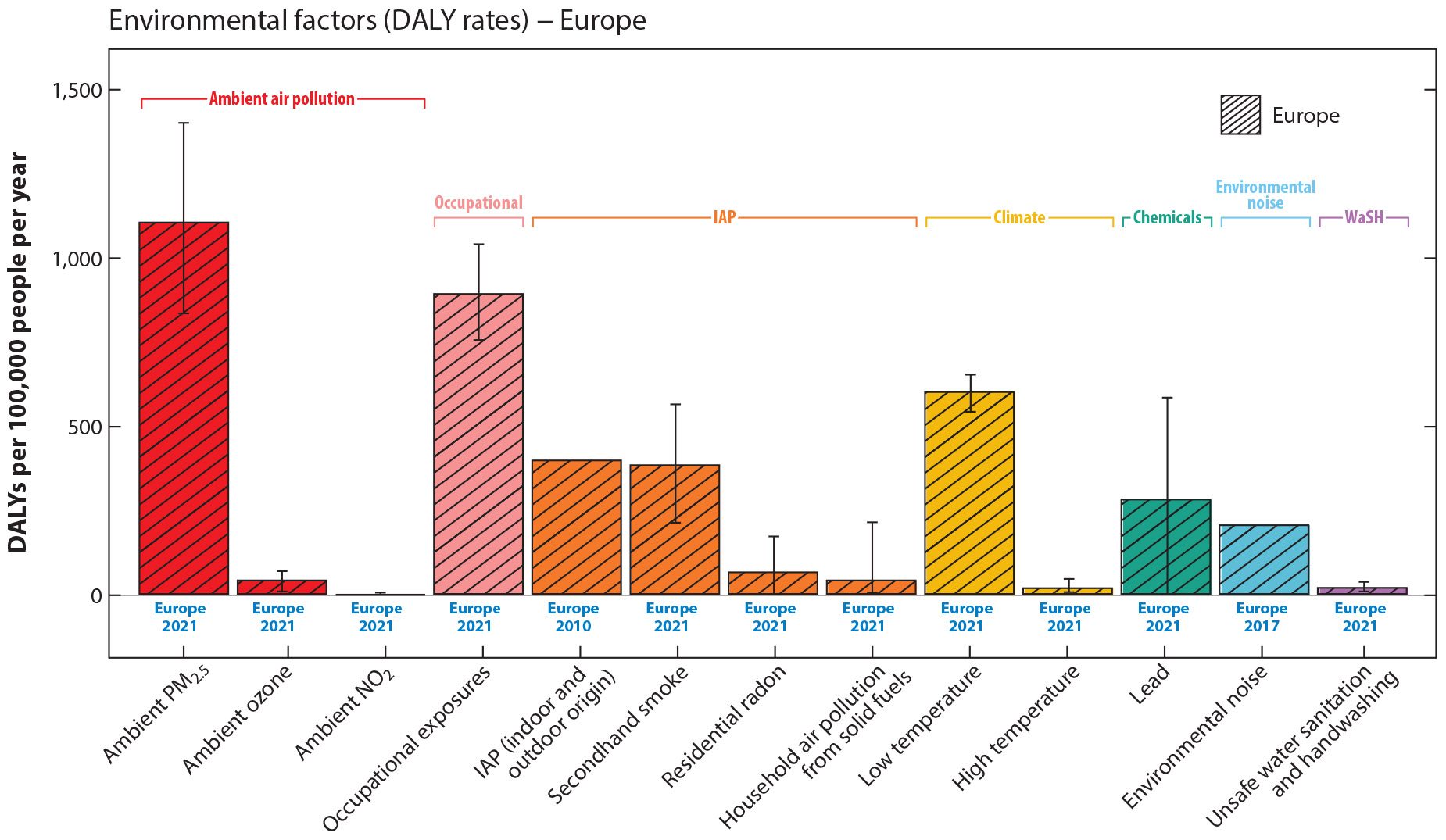
Annual DALYs lost per 100,000 people (all ages) in Europe attributable to environmental risk factors covered within this review. DALY rates from ambient air pollution (*red*) and IAP (*orange*) are sourced from the GBD 2021 project data (9), although DALY rates attributable to IAP (from indoor and outdoor origin) were estimated for 26 European countries for 2010 from the 2016 HealthVent study (3). Note that secondhand smoke DALY rates from GBD 2021 when restricting the geographic area to the European Union countries are reduced to 268. DALY rates for the occupational exposures (*pink*) and WaSH (*purple*) categories are from GBD 2021 (9). The chemicals (*turquoise*) category includes DALY rates attributable to lead exposures from GBD 2021. The climate (*yellow*) category includes DALY rates attributable to low and high temperatures from GBD 2021 (9). The environmental noise (*blue*) category includes environmental noise–attributable DALYs due to exposures from road, rail, aircraft, and industrial sources, estimated for 33 countries in the EEA (not including Turkey) for 2017 as reported in the EEA 2020 report (24). When standardized population rates were not given, total numbers were standardized by the estimated regional population for the corresponding years. Uncertainty intervals are included where available. Abbreviations: DALYs, disability-adjusted life years; EEA, European Economic Area; GBD, Global Burden of Disease; IAP, indoor air pollution; PM_2.5_, particulate matter with diameters ~2.5 μm and smaller; WaSH, unsafe water, sanitation, and hygiene.

The evidence summarized in this review has demonstrated that environmental risk factors substantially contribute to the global burden of disease, as captured within the GBD study and other sources. Efforts to estimate the impact of environmental exposures suggest that 15–25% of global disease burden is attributable to environmental risk factors. We made comparisons between DALYs and mortality rates attributable to key environmental risk factors included in the GBD study, as well as from climate change, chemicals, IAP, and environmental noise, using data from other sources where global or regional comparisons were reasonably possible (estimates for IAP and environmental noise were available only for Europe) (Figures 1 and 2).

Globally, ambient air pollution exposure (PM_2.5_) is the largest contributor to the environmental disease burden (DALYs), due in particular to its impact on mortality (Figure 1), followed by HAP (from solid fuel burning), occupational exposures, and unsafe WaSH. Focusing specifically on contributions to mortality, ambient PM_2.5_ air pollution still leads, followed by HAP from solid fuel burning, lead, and low temperatures. Unmitigated climate change may exacerbate exposures such as ambient PM_2.5_ over time, potentially worsening attributable health burdens (not illustrated in Figure 1).

Moreover, our comparisons indicate that other exposures not currently captured within the GBD, such as environmental noise or IAP, also account for a sizeable number of DALYs in Europe (Figure 2). The population-standardized global environmental disease burden may also be relatively higher than what is currently estimated within European countries, given the geographic variability in exposure distributions and other underlying factors affecting vulnerability to exposures. This lack of information emphasizes the urgent need to fill global data and evidence gaps for many unquantified environmental risk factors worldwide.

## Figures and Tables

**Table 1:** Global disease burden attributable to environmental risk factors in the Global Burden of Disease Study [GBD 2021 (33)]

Risk factor	TMREL	Deaths in 2021 (millions)			% of DALYs in 2021			% change in DALYs (2010–2021)		
		Central estimate	Upper bound	Lower bound	Central estimate	Upper bound	Lower bound	Central estimate	Upper bound	Lower bound
**Environmental/occupational risks**		**12.8**	**14.6**	**10.9**	**14.5**	**16.2**	**12.9**	−**14.88**	−**9.55**	−**20.26**
Ambient particulate matter pollution (PM_2.5_)	2.4–5.9 μg/m^3^ annual mean	4.72	5.80	3.48	4.2	5.2	3.0	21.91	33.89	11.60
HAP from solid fuels (PM_2.5_)	2.4–5.9 μg/m^3^ annual mean	3.11	5.19	1.90	3.9	5.7	2.6	−30.85	−18.29	−40.65
Lead exposure	0.016 μg/dL blood	1.54	3.22	−0.18	1.2	2.4	0.0	12.52	20.35	2.60
Low temperature	Temperature associated with the lowest mortality for all included causes	1.48	1.64	1.30	0.9	1.0	0.8	10.19	19.43	1.70
Occupational risks^a^	Zero exposure	1.44	1.60	1.30	2.7	2.9	2.5	−1.93	1.51	−5.53
Secondhand smoke	Zero exposure	1.29	1.90	0.68	1.2	1.8	0.6	−5.95	0.83	−11.68
Unsafe water source	Access to high-quality piped water	0.80	1.22	0.37	1.5	2.0	0.8	−45.39	−38.12	−52.44
Unsafe sanitation	Access to a sanitation facility with sewer connection or septic tank	0.59	0.83	0.41	1.1	1.4	0.9	−48.85	−41.89	−55.17
Ambient ozone pollution	29.1–35.7 ppb [highest seasonal (6-month) average of 8-h daily maximum]	0.49	0.84	0.11	0.3	0.5	0.1	25.10	38.79	15.19
No access to handwashing facility	Access to a handwashing facility with soap (bar, liquid, or powder/detergent), water, and wash station (either permanent or mobile)	0.45	0.97	−0.12	0.8	1.8	0.0	−46.27	−38.02	−55.64
High temperature	Temperature associated with the lowest mortality for all included causes	0.44	0.62	0.29	0.5	0.6	0.3	−33.60	−19.70	−45.18
Residential radon	O Bq/m^3^	0.08	0.21	−0.04	0.1	0.2	0.0	14.30	24.75	4.41
Nitrogen dioxide pollution	4.6–6.2 ppb annual mean				0.01	0.02	0.00	−20.61	−10.16	−45.71

Abbreviations: Bq, becquerel; DALY, disability-adjusted life year; HAP, household air pollution; ppb, parts per billion; TMREL, theoretical minimum risk exposure level.

Upper and lower bounds represent 95% uncertainty intervals around the central estimates.

a Occupational risks include occupational injuries; occupational ergonomic factors; occupational exposure to particulate matter, fumes and gases, noise, asthmagens, and carcinogens (asbestos, arsenic, benzene, beryllium, cadmium, chromium, diesel engine exhaust, formaldehyde, nickel, polycyclic aromatic hydrocarbons, silica, sulfuric acid, trichloroethylene).
